# Editorial: The heterogeneity in COPD phenotypes

**DOI:** 10.3389/fmed.2022.982121

**Published:** 2022-08-15

**Authors:** Naoya Tanabe, Chin Kook Rhee, Hye Yun Park, Masaru Suzuki

**Affiliations:** ^1^Department of Respiratory Medicine, Graduate School of Medicine, Kyoto University, Kyoto, Japan; ^2^Division of Pulmonary and Critical Care Medicine, Department of Internal Medicine, Seoul St. Mary's Hospital, College of Medicine, The Catholic University of Korea, Seoul, South Korea; ^3^Division of Pulmonary and Critical Care Medicine, Department of Medicine, Samsung Medical Center, Sungkyunkwan University School of Medicine, Seoul, South Korea; ^4^Department of Respiratory Medicine, Faculty of Medicine, Hokkaido University, Sapporo, Japan

**Keywords:** COPD, phenotype (mesh), asthma, spirometry, bronchiectasis, emphysema

Chronic obstructive pulmonary disease (COPD) is diagnosed simply based on the presence of not fully reversible airflow limitation on post-bronchodilator spirometry. However, there are substantial heterogeneity in the clinical manifestations and outcomes despite a similar degree of airflow obstruction (1). The vigorous work to identify the heterogeneity of the disease and to select the treatment with the most favorable response by clinical phenotype has been under progress (2). Cigarette smoke is a leading cause of COPD, but never smokers can also develop COPD in associations with occupational exposure to toxic gases and biomass fuels, and a history of asthma, tuberculosis or respiratory infections in childhood. Accordingly, in addition to a rapid lung function decline after adulthood, abnormal lung growth with subsequent normal lung function decline leads to COPD (3).

As another type of spirometry abnormality, there is a growing interest in preserved ratio-impaired spirometry (PRISm), characterized by lower forced expiratory volume in 1 s (FEV_1_) with a preserved FEV_1_/Forced vital capacity ratio. The concept of PRISm is related to the older concept of so-called non-specific pulmonary function pattern (4). A large cohort recently showed that the PRISm presence is associated with poor clinical outcomes (5). Iyer et al. showed that over a median follow-up period of 3 years, 15% of subjects with non-specific pulmonary function transitioned to airflow limitation while 64% remained the non-specific pattern. Subsequently, Wan et al. showed that over 5 years, 25% of subjects with PRISm at baseline transitions to COPD at follow-up spirometry (6). However, the majority also remains stable overtime. Thus, whether the PRISm precedes COPD is still controversial.

From a structural perspective, imaging parameters using chest computed tomography (CT) has been widely studied, which showed that airway disease dominant and emphysema dominant COPD have distinct clinical features (7–9). Moreover, bronchiectasis and incidental fibrotic change also affect clinical outcomes in COPD (10). Many reports have increased the understanding of relevant phenotypes with distinct outcomes and simultaneously urged us to identify treatable traits such as inhaled corticosteroid (ICS) responders regardless of phenotypes to perform individualized disease management (11). Particularly, some patients with COPD have an inflammatory pattern with increased eosinophils and accumulated studies have shown that blood eosinophil counts predict the magnitude of the effect of ICS. Furthermore, in individuals without COPD, higher blood eosinophil counts are associated with increased risk of the subsequent development of COPD (12). Apart from eosinophilic COPD, concomitant asthma or a prior history of asthma could coexist in COPD patients, which may be categorized as asthma-COPD overlap (ACO) (13, 14).

Under these circumstances, we have launched this Research Topic entitled “The heterogeneity in COPD phenotypes.” In this topic, six articles have been published. Emphysema COPD phenotype is associated with a rapid FEV_1_ decline, exacerbation, and mortality (15). Gomes et al. established a novel method for emphysema quantification on CT. They showed that the ratio of emphysema volume to predicted lung volume might reflect the severity of emphysema more accurately than the ratio of emphysema volume to measured lung volume because lung volume increased as the diseases progresses due to lung hyperinflation.

The coexistence of bronchiectasis and COPD is increasingly recognized as bronchiectasis-COPD overlap (BCO). Kim S. H. et al. explored the impact of COPD on health-related quality of life (QoL) using the Bronchiectasis Health Questionnaire (BHQ) in bronchiectasis patients using data of the Korean Multicentre Bronchiectasis Audit and Research Collaboration (KMBARC) registry. Of 598 bronchiectasis patients, 226 (37.8%) had COPD and showed that dyspnea [modified Medical Round Council scale (mMRC) ≥ 2], depression [Patient Healthy Questionnaire 9 (PHQ-9)], and fatigue [Fatigue Severity Score (FSS)] were associated with decreased QoL (BHQ score <57) in bronchiectasis patients with COPD. Moreover, Lei et al. examined data from the Acute Exacerbation of Chronic Obstructive Pulmonary Disease Inpatient Registry (ACURE) in China to compare clinical features between patients with ACO, BCO, and their coexistence (ABCO). Of 4,813 patients with COPD, 7.02, 10.22, and 1.31% were categorized into ACO, BCO, and ABCO phenotypes. Notably, the ABCO phenotype was characterized by younger age, more prior-allergic episodes, respiratory failure, anxiety and depression than the other phenotypes.

In terms of ACO, Jo et al. reported racial differences in the prevalence and clinical features of ACO using two datasets from the Korean COPD Subgroup Study (KOCOSS) and the COPD Genetic Epidemiology (COPDGene) study. They showed that the prevalence of ACO, defined as a bronchodilator response >15% and 400 ml and/or blood eosinophil count ≥300/μl, was 21.4, 17.4, and 23.8 in non-Hispanic white (NHW), African American (AA), and Asian. Asian patients with ACO are more likely to be older, male, and have less smoking history than NHW and AA patients with ACO. Moreover, in patients with ACO, the risk of moderate-to-severe exacerbation was lower in ICS users than non-ICS users.

Kim Y. et al. used data from the KOCOSS cohort and showed that more severe air-trapping assessed as the ratio of residual volume to total lung capacity (RV/TLC) was associated with an increased risk of exacerbations. Interestingly, when patients were divided into those with and without triple inhaler therapy (ICS/LABA/LAMA), the association of higher RV/TLC with higher risk of exacerbations was found only in those without tripe inhaler therapy. The nullification of the association between RV/TLC and exacerbation in those with tripe therapy suggests that triple therapy could reduce the risk of exacerbation in patients with greater air-trapping.

Finally, He et al. analyzed the publicly available dataset of the English longitudinal study of aging. Of 6,616 subjects, 12.9% were classified into severe PRISm, defined as both FEV_1_ and FVC% predicted <80%, and 7.5% were classified into mild (non-severe) PRISm. The subjects with severe PRISm had more phlegm, wheezing, dyspnea, chronic bronchitis and emphysema than those with mild PRISm. Moreover, severe PRISm, but not mild PRISm, was more closely associated with the development of moderate to severe COPD (FEV_1_ %predicted <80%) and higher mortality than normal spirometry.

In summary, these 6 manuscripts have confirmed the considerable heterogeneity in clinical manifestations in subjects with COPD and those with PRISm. Emphysema, bronchiectasis, concomitant asthma, and air-trapping are all associated with distinct clinical features in patients with COPD. Based on these findings, we believe that future studies should investigate whether pharmacological and/or non-pharmacological interventions including pulmonary rehabilitation and lung volume reduction could improve clinical status and outcomes in patients with distinct features to establish a more personalized management of the disease.

## Author contributions

All authors listed have made a substantial, direct, and intellectual contribution to the work and approved it for publication.

## Conflict of interest

The authors declare that the research was conducted in the absence of any commercial or financial relationships that could be construed as a potential conflict of interest.

## Publisher's note

All claims expressed in this article are solely those of the authors and do not necessarily represent those of their affiliated organizations, or those of the publisher, the editors and the reviewers. Any product that may be evaluated in this article, or claim that may be made by its manufacturer, is not guaranteed or endorsed by the publisher.
